# Supplementary material for: Acceptance and compliance with micronutrient powder (MNP) among children aged 6–23 months in northern Nigeria
Source: PLOS Glob Public Health. 2022 Oct 17;2(10):e0000961. doi: 10.1371/journal.pgph.0000961 (PMC10022258; doi:10.1371/journal.pgph.0000961)
Supplement: S1 Dataset — (ZIP) [file pgph.0000961.s008.zip › S8_Dataset/Direct Observations.zip!/Direct Observations/180615_K_BK_SU_1_DO.pdf]

1.1.1 Instrument F –Direct Observation Form

<<Instrument F Direct Observation Form>>
**PI Name:** PragyaMathema,Stephen Kodish<br>**Study Title:** Formative research to develop an infant and young child feeding (IYCF) intervention with micronutrient powder (MNP) for children aged 6 – 23 months<br>**PI Version/Date:** Version1.0/January 24, 2015

Date:18/06/2015 Location: Goru Chindo

Data Collector: Samira Usman

Main goals of this direct observation are to answer the following questions:<br><br>1. In <u>this</u> household, what would be the best way(s) to give nutritional supplements to <u>young children</u>? Why?<br><br>2. In <u>this</u> household, describe the food sharing that you observe throughout the meal. Are there any <u>predictable</u> patterns?<br><br>3. In <u>this</u> household, who is favored and how is this favoritism expressed during meal preparation and eating?

Household Composition Table (Fill out <u>as</u> you do data collection)

INITIALS	SEX (M/F)	AGE (estimated)	Relationship to household	Description
MM	F	20yrs	Mother	Red wrapper and pink tie with average height
BBI	M	5yrs	Cousin brother living in the house	Wearing brown trousers
BB2	M	11 months	Baby	Green short
BG	F	2yrs	Daughter	Milk pant
BG 2	F	7yrs	Cousin sister living in the same compound	Black gown and green cap
MI	F	53yrs	Mother in-law	Blue wrapper and head tie
WT	F	31yrs	Senior brothers wife	Red and blue wrapper, top and head tie (Ankara)
TM	M	18yrs	The son of a brothers wife (cousin)	Black jeans and red short

FI	M	60yrs	Father in-law	Milk long gown, grey hair (jalabiya)

TIME	ACTOR INITIALS	RECIPIENT INITIALS	WHAT HAPPENED	FOOD	Reactivity
10:00	MM	BB2	Giving the baby water in the silver cup	water	
10:05	BG		Eating tuwo rice with baobab leaves soup	Tuwo and dried baobab soup	
10:15	BB2		Crowing and playing alone		
10:20	BB1		PLAYING AROUND THE HOUSE		
10:25		Bb2	Mother breastfeeding the baby		
10:30	BG		Playing around the house		
10:35	MM		Mother sweeping the house		
10:40	MM	BG	Beating the baby girl to put on her pant		R
10:45	MM	BB2	Baby crying and mother took him and give him water	water	
10:50	MM	BG	Mother combing the baby girl hair		
10:55	MM		Brother going out to the next house		

TIME	ACTOR INITIALS	RECIPIENT INITIALS	WHAT HAPPENED	FOOD	Reactivity
11:00	MM	BB2	Mother breast feeding the baby		
11:05	MM	BB2	Mother backing the baby		
11:10	MM		Giving the remaining food to the goats in the house with the same bowl		
11:15	MM	BB	Mother bringing out a mat and wrapper, lay the wrapper on the mat and then keep the baby, the baby is sleeping		
11:20	MM	BB2	The baby is crying and the mother is breast feeding him lying down with him, so he slept again		
11:25	BB2		Still sleeping		
11:30	MM	BB2	The baby woke up and starts crying and the mother took him and breast feed him		
11:35	MM	BB2	Mother giving the baby pap in a small cup and add MNP powder he take small and leave the remaining	Pap made of millet	R
11:40	MM	BB2	Mother playing with the baby		
11:45	MM	BB2	Mother breast feeding the baby		
11:50	MM	BB2	Mother giving the baby water		
11:55	BB2	BB2, BG1	Eating rice and as well giving the baby and the BG1	Rice and oil	
12:00	BB2		Baby crawling and playing around		
12:05	MM	BB2	Baby is having diarrhea and the mother washing his body and pant and picking the one on the ground with broom and packer		
12:10	MM		Mother washing clothes		
12:15	MM	BB2	Baby is crying and the mother took him and breast feed him		

TIME	ACTOR INITIALS	RECIPIENT INITIALS	WHAT HAPPENED	FOOD	Reactivity
12:20	BB2		Baby playing with plastic plate		
12:25	MM	BB2	Mother backing the baby because he is crying		
12:30	MM		Still washing clothes		
12:35	MM	BB2	Mother keeping the baby down and he is playing		
12:40	BG2		Fetching water from the tap outside the house		
12:45	BG2	BB2	The girl carries the baby outside the house		
12:50	MM	BB2	The baby is crying and the mother took him from the girl and breast feed him		
12:55	BB2		The baby is sleeping		
1:00	MM		Washing clothes		
1:05	BB2		The baby still sleeping		
1:10	BB2		The baby still sleeping		
1:15	BB2		The baby still sleeping		
1:20	BB2		The baby still sleeping		
1:25	BB2		The baby still sleeping		
1:30	MM	BB2	Baby just wake up and starts crying and the mother took him and gave him pap	pap	
1:35	MM	BB2	Mother is breastfeeding the baby		
1:40	MM	BB2	Still breast feeding the baby		
1:45	Bb2		Baby is sleeping		
1:50	BB2		Baby still sleeping		
1:55	MM		Mother washing clothes		

TIME	ACTOR INITIALS	RECIPIENT INITIALS	WHAT HAPPENED	FOOD	Reactivity
2:00	BB2		Still sleeping		
2:05	BB2		Still sleeping		
2:10	BB2		Sleeping		
2:15			Sleeping		
2:20			Sleeping		
2:25	BG		The small girl is eating boiled kenaf giving to her by a woman next to their house		
2:30	BB2		Baby still sleeping		
2:35			sleeping		
2:40			Sleeping		
2:45	BB2		Sleeping		
2:50	MM	BG	Mother is bathing the small girl		
2:55	MM		Mother carry a bucket of water and enter the toilet		
3:00	BB2		Baby still sleeping		
3:05	MM	BB2	Baby wakes up crying and the mother took him and bath him in a rubber, then give him water to drink in a cup; then the mother sat down and breast feed the baby		
3:10			Still breast feeding the baby		
3:15			Breast feeding the baby		
3:20			Baby playing around		
3:25	BB2	MM	Baby crying and the mother brought kenaf (rama) for the child and he		

TIME	ACTOR INITIALS	RECIPIENT INITIALS	WHAT HAPPENED	FOOD	Reactivity
			refused to eat		
3:30	MM	BB2	The baby is still crying, the mother feed him with pap in a cup and he drank all	pap	
3:35	BB2		Baby sitting on a mat scratching his body		
3:40	BB2		Baby playing with his sister		
3:45	MM	BB2	Mother putting the baby on her lap with his leg open and he starts passing stool		
3:50	MM	BB2	Mother washing the baby body and find sand and pack the stool on the ground		
3:55	BG		Small girl sleeping in her ground mother in the compound		
4: 00	MM	BB2	Mother feeding the baby with white rice and oil		
4:05	MM	BB2	Mother gives the baby water and then breast feed him		
4:10	BB2		Baby playing with the rice in the bowl and picking those on the ground and eating		
4:15	BB2, MM	BB2	Baby starts crowling and following the mother crying and she carried him sit down and breast feed him		
4:20	MM	BB2	Mother sleeping and the baby is breast feeding		
4: 25	BB2		Baby is playing		
4:30	MM	BB2	Baby is crying and the mother lie down with the baby breast feeding him		
4:35	BB2		Baby is sleeping		
4:40	MM	BB2	Both the mother and the baby are sleeping, baby breast feeding		
4:45	BB2		Baby still sleeping		
4:50	MM	BB2	Baby wakes up and the mother give him water		

TIME	ACTOR INITIALS	RECIPIENT INITIALS	WHAT HAPPENED	FOOD	Reactivity
4:55	BB2		Baby is playing on his mothers back		
5:00	MM	BB2	Mother is lying down sleeping and the baby is breast feeding		
5:05	MM	BB2	Mother is breast feeding the baby and he is sleeping		
5:10	MM	BB2	Mother breast feeding the baby		
5:15	MM	BB2	Mother breast feeding the baby		
5:20			Baby and mother still sleeping		
5:25			Baby and mother still sleeping		
5:30			Baby and mother still sleeping		
5:35	MM	BG	Mother went into the room and bring pap to the girl in a cup and the girl is drinking it her self	pap	
5:40	MM	BG	Baby girl says she want to drink water and the mother give her the water		
5:45	MM		Mother is setting fire		
5:50	MM		Washing the pot and then add water into the pot and set fire in the presence of her in-law		
5:55	MM	BB2	Baby wakes up and the mother gives him water and backs him. Mother set fire and add water in another pot, add palm oil and onions, tomatoes and pepper with grinded locust beans magi, salt, dried ginger and then closes the pot.	water	
6:00	TM		Came in the house with a bike and took a jerican and went outside to fetch water 3 times emptying the water into a big pot		
6:05	MI		Mother in-law is preparing gruel with the boiling water that her daughter in-law put on the fire. She		

TIME	ACTOR INITIALS	RECIPIENT INITIALS	WHAT HAPPENED	FOOD	Reactivity
			add the boiling water into the watery pastry, it turns into gruel. She add small quantity of cold water on top of the gruel and take it to her room		
6:15	WT		The brother's wife wash the rice with water and add into the boiling pot and close it		
6:20	MM	BB2	Mother keep the baby on the ground and give him water and the baby is still playing	water	
6:25	BB2		Baby still playing		
6:30	BG2		The 7 year old girl is cutting the vegetables in a calabash		
6:35	BB2		Baby playing		
6:40	BG	BB2	A two year old girl is drinking pap and giving to her brother to drink	Pap	
6:45	MM	BB2	Mother is playing with the baby		
6:50	MM	BB2	Mother is breast feeding her baby		
6:55	MM	BB2	Mother is backing the baby		
7:00	MM	BB2	Mother backing the baby		
7:05	BB2		Baby is crying		
7:10	MM	BB2	Mother bring him down to breast feed him		
7:20	BG2		The young girl washes the spinach with water and add into the pot and closes it	Spinach soup	
7:25	WT		The brother's wife is pounding the rice in the pot until it is very soft and she closes the pot	Tuwo rice	
7:30	WT		She again open the pot and re open it again and serve to the members of the family		

7:35	MM	BB2	Mother is backing the baby		
7:40	BB2		Baby sleeping on his mother back		
7:45	WT		The senior brother's wife is serving the food. The husband has his bowl, her children in one big bowl. Whoever wants to eat fetch from the bowl, she has her own bowl.<br>Her brother's wife (mother of the baby) has her own bowl with her children, her husband has his own bowl.<br>Mother in law has her own bowl, father in law has his own bowl		Tuwo rice and spinach soup
7:50	MM<br>BG	BB2	The baby wakes up and the mother give him water and breast feed him and keep him on the ground playing with his sister		
7:55	TM		Older cousin brother came into the house and fetch food and went out with it		
	BG2		The young girl is eating a lone		
	BB1		The young boy is eating alone		
	BG1		The two yr old is eating alone		
	MI		Mother inlaw is eating alone		
8:05	MM	BB2	Baby is crying and mother is breast feeding the baby	Tuwo rice and spinach soup	
8:10	MM	BB2 BG	Mother is eating and giving to her baby and 2 years old is eating with them again		

Primary Questions (Complete <u>after</u> you complete data collection):

From what you have seen observing THIS HOUSEHOLD, please answer the following questions in detail:

Question	Write your detailed response here
1. In this household, what would be the best way(s) to give nutritional supplements such as an MNP to young children to ensure compliance?<br><br>Why?	The best way to give MNP in this household is through the mother because the mother is fully in-charge of taking care of the baby
2. In this household, who is favored during meal preparation or feeding?<br><br>How is this favoritism expressed?	The baby because he is always protected by his mother and she is eating he is on her lap and she is given him the food little by little
3. How could we promote the MNP supplement for this family that would attract their attention?<br><br>Who would they listen to?	Through their husbands because the wife accepts and obey whatever her husband says i.e his decision is final<br><br>Secondly, the in-law as I can see they so much respect them and obey their instruction
4. In this household, what would be the best ways to promote the MNP but limit how much it would be shared?<br><br>Why?	Through proper sensitization of the product to the caregivers because as I can see they do not have enough information about the product
5. What other issues and concerns did you see with this family that are important for us to consider with regard to introduction of an MNP?<br><br>Why?	The issues and concern I saw with regards to this family that are important with regard to the introduction of MNP is the administration of MNP because when she put the MNP in the cup of pap the child could not finish it and she left it there, at long last the cup fell and the pap pour away, carelessness is my concern. Hygiene is also another concern, although the part of the house where I stood is to some extend clean but the compound in which animal are rear is close to where food is cooked, children passing stools and urinating wherever they want. The household is an

	extended family
6. Based on your experience observing this child today, do you believe reactivity negatively impacted your ability to see the food-related behaviors of the child?<br><br>Please explain your answer in detail.	I do not think reactivity negatively impacted on my ability to see the food related behavior of the child because with time they even tend to forget about my presence and continue with their activities.<br>My only concern is during the MNP administration, she was looking at me and facing me so that I can see that she is administering the MNP and even asking me about the product and I pretended I don't know what it is. I think maybe they were told about our coming.
